# Real-World Molecular Testing and Treatment Patterns in Brazilian Patients with Newly Diagnosed Locally Advanced or Metastatic NSCLC

**DOI:** 10.6061/clinics/2020/e1777

**Published:** 2020-10-05

**Authors:** Eduardo Cronemberger, Clarissa Baldotto, Felipe Marinho, Pedro De Marchi, Luiz Henrique Araújo, Fabio Franke, Paulo Salles, Aknar Calabrich, Thaís Almeida, Marcelo Graziano Custodio, André Santa Maria, Marcelo Horacio Pereira, Gilberto Castro

**Affiliations:** I Centro Regional Integrado de Oncologia (CRIO), Fortaleza, CE, BR; II Oncologia, Instituto D’Or de Pesquisa e Ensino, Rio de Janeiro, RJ, BR; III Hospital de Cancer de Pernambuco, Recife, PE, BR; IV Hospital de Cancer de Barretos, Barretos, SP, BR; V Instituto Nacional de Cancer (INCA), Rio de Janeiro, RJ, BR; VI Associacao Hospital de Caridade Ijui, Ijui, RS, BR; VII Instituto Mario Penna, Belo Horizonte, MG, BR; VIII Clinica AMO, Salvador, BA, BR; IX Instituto de Oncologia do Parana, Curitiba, PR, BR; X AstraZeneca, Cotia, SP, BR; XI Instituto do Cancer do Estado de Sao Paulo (ICESP), Sao Paulo, SP, BR

**Keywords:** Lung Neoplasms, Non-Small-Cell Lung Cancer, Adenocarcinoma, EGFR

## Abstract

**OBJECTIVES:**

To evaluate the molecular testing and treatment patterns in a retrospective cohort of newly diagnosed treatment-naïve patients with locally advanced or metastatic non-small-cell lung cancer (NSCLC).

**METHODS:**

This is an observational retrospective cohort study conducted across 10 cancer centers in Brazil. Treatment-naïve patients with locally advanced or metastatic NSCLC were enrolled from January to December 2014. The following data were collected from the medical records of patients from diagnosis until the last record (death, loss to follow-up, or the end of the maximum follow-up period): demographics; medical history; smoking status; disease characteristics; previous treatments; and molecular testing patterns and results. The overall survival (OS) was also estimated.

**Results:**

A total of 391 patients from 8 different Brazilian states were included, with a median age of 64.1 years (23.7-98.7), with most patients being males (60.1%). The smoking status of 74.2% of patients was a ‘former’ or ‘current smoker’. Stage IV NSCLC at diagnosis was observed in 82.4% of patients, with 269 of them (68.8%) presenting adenocarcinoma (ADC). Among the stage IV ADC patients, 54.0% were referred for molecular testing. Among the patients with an available epidermal growth factor receptor (EGFR) mutation status, 31 (24.0%) were EGFR-positive. The first-line treatment was a platinum-based chemotherapy for 98 patients (25.1%), while non-platinum-based regimens were used in 54 patients (13.8%). OS data were available for 370 patients, with a median OS of 10.8 months. Never smokers had a significantly higher median OS *versus* current or former smokers (14.6 *versus* 9.1 months; log-rank *p*=0.003). Among the patients for whom molecular testing data were available, those with EGFR-positive results had a longer median OS (34.6 *versus* 12.8 months; log-rank *p*=0.003).

**Conclusion:**

Our findings provide relevant information for prescribers and policy decision-makers by highlighting the unmet needs of patients and the importance of molecular testing in newly diagnosed locally advanced or metastatic lung adenocarcinoma. We also highlight the respective EGFR-tyrosine kinase inhibitor treatment when the result is positive and the areas in which further efforts are required to grant access to effective treatment.

## INTRODUCTION

Lung cancer is the global leading cause of cancer deaths, with 2 million cases and 1.7 million deaths projected in 2018, according to the Global cancer statistics of 2018 (1). The Brazilian National Cancer Institute estimates that among cancers, lung cancer has the second highest incidence rate among men (16.99/100,000) and the fourth highest, among women (11.56/100,000) in the country, with 30,000 new cases expected per year for the 2020-2022 period (2). In Brazilian studies, non-small-cell lung cancer (NSCLC) represents 80-92% of all lung cancer cases, with approximately 70% of patients presenting locally advanced or metastatic disease (stages III and IV, respectively) at diagnosis (3).

In recent years, the most significant advance in lung cancer management has been the evolving knowledge regarding the biology of NSCLC, particularly with regard to genetic alterations that behave as malignancy drivers and directly impact the prognosis and treatment response. Tumors expressing some molecular mutations (e.g., activating alterations in the epidermal growth factor receptor (EGFR), Anaplastic lymphoma kinase (ALK), and c-ros oncogene 1 (ROS1) genes) are suitable for specific therapeutic approaches associated with survival improvement. Therefore, clinical guidelines state that NSCLC patients, especially those with a non-squamous histology, should have access to molecular testing in order to identify those who can benefit from targeted therapies (4).

Nevertheless, lung cancer patients in Brazil still face barriers to access genetic testing. There is also a paucity of observational data regarding the frequency of specific driver mutations, markedly within the Brazilian public health care system. A recent review reported that less than half of the Brazilian patients enrolled in a survey conducted in 2014 were tested for EGFR activating mutations (EGFRms), with the frequency of testing being even lower among patients receiving public health care services (3). The same review summarized the distribution data of some molecular markers in Brazilian patients and found that the frequency of EGFRms (approximately 25%) is similar to that in other Latin American countries (3).

Targeted therapies, including EGFR-tyrosine kinase inhibitors (EGFR-TKIs), have been approved in Brazil and used in front-line settings in NSCLC patients whose tumors harbor EGFR activating mutations. The USA National Comprehensive Cancer Network (NCCN) guidelines recommend EGFR-TKIs as a first-line therapy in advanced/metastatic NSCLC EGFRm-positive patients (5). However, cytotoxic chemotherapy is still widely used, mainly in the public health care system, despite the commercial availability of EGFR-TKIs. A better access to molecular testing to determine the EGFR mutational status and a shift from a chemotherapy-based regimen to an oral targeted therapy may likely impact the current standard of care among these patients in Brazil. Thus, there is a need to understand the local real-world testing patterns, treatment patterns, and outcomes among newly diagnosed NSCLC patients. Here, we aimed to evaluate the molecular testing and treatment patterns in a retrospective cohort of newly diagnosed patients with locally advanced or metastatic NSCLC, who were treatment-naïve.

## MATERIAL AND METHODS

### Study Design

This is an observational retrospective cohort study conducted in 10 cancer centers in Brazil. Patients with locally advanced (not amenable to local therapies with curative intent) or metastatic NSCLC who were treatment-naïve were consecutively identified in participating sites from January 1^st^ to December 31^st^, 2014. Data were collected from the medical records from diagnosis until the last record (death, loss to follow-up, or at the end of the maximum follow-up of 36 months, in December 2017). Data from patients who were alive at the end of the study follow-up were censored and no further follow-up was required.

### Population

Patients were deemed eligible if they were male or female patients ≥18 years of age, newly diagnosed with locally advanced (not amenable to local therapies with curative intent) or metastatic NSCLC, and who were treatment-naïve. The patients who were enrolled in clinical trials that prohibited any participation in this observational study were excluded.

### Variables and outcomes

The following data were collected from the medical records: demographics; past medical history; smoking status; date of diagnosis; extent of disease, based on the physician’s interpretation; tumor characteristics, such as histology and stage (American Joint Committee on Cancer (AJCC), seventh edition); previous treatments (chemotherapy, radiation therapy, or targeted therapies); self-reported ethnicity; and cancer family history. The frequency of molecular testing patterns and molecular findings (mutation status and type) were determined, along with the characterization of the treatment patterns (chemotherapy/targeted therapy, surgery, radiation therapy, and palliative or supportive care) and institutional profile (public *versus* private). The overall survival was also estimated.

### Statistical analysis

Patients, their tumor characteristics, and treatment patterns were presented using descriptive statistics. Continuous variables were summarized based on their central tendency and dispersion measures. The distribution of data was assessed using the Kolmogorov-Smirnov test and quantile-quantile (Q-Q) plot. To make comparisons of proportions between specific groups, the Chi-Square test, the continuity correction test, and the Fisher test were used when appropriate, depending on the minimum expected value in the case of square crosstabs. Quantitative variables were compared between specific groups using the parametric Student's t-test and the non-parametric Mann-Whitney test. To establish predictors of binary outcomes, multiple binary logistic regression was used, by analyzing the odds ratio and the 95% confidence intervals (95% CIs). To describe and plot the overall survival, the Kaplan-Meier method was used to calculate the median survival, and comparisons of survival curves were performed using the Log-Rank test method. The Statistical Package for the Social Sciences (SPSS) software version 22.0 was used and any *p*-value<0.05 for a two-tailed test was considered statistically significant.

In this retrospective cohort, precision estimates were calculated considering a categorical endpoint (i.e., the percentage of total newly diagnosed NSCLC patients undergoing molecular testing, the primary objective). Scenarios were tested with regard to the cohort size and the corresponding precision estimates for the outlined measure. For a cohort size of 400 patients, the precision estimate for a 50% testing frequency was 45.0-55.0% (95% CI based on the binomial Clopper-Pearson exact method).

### Ethical conduct

The study was performed in accordance with the ethical principles that are consistent with the Declaration of Helsinki, International Conference on Harmonisation Good Clinical Practice (ICH GCP), and the applicable national legislation. The final protocol of the study was approved by the Ethics Committee (EC) of each participating site, under the following approval numbers: Hospital Erasto Gaertner n. 2.328.101; Clínica AMO n. 2.020.772; Instituto COI n. 1.971.437; Instituto do Câncer do Estado de São Paulo (ICESP-FMUSP) n. 2.079.850; Centro Regional Integrado de Oncologia (CRIO) n. 1.956.955; Fundação PIO XII n. 1.885.890; Hospital de Caridade Ijuí n. 1.959.243; Instituto Nacional do Câncer José Alencar n. 2.169.046; Sociedade Pernambucana de Combate ao Câncer n. 1.975.285; Associação Mario Penna n. 2.255.393. The study procedures were initiated only after the final ethical approval.

An interim analysis of this trial has been presented in WCLC 2018, in a poster section as follows: P3.01-12 EGFR Mutation and Targeted Therapies: Difficulties and Disparities in Access to NSCLC Treatment in Brazil (6).

## RESULTS

### Patient population

A total of 391 patients from 8 different Brazilian states were included in the study cohort. Their main clinical and demographic characteristics are described in Table 1. Their median age was 64.1 years (range, 23.7-98.7), and most patients were male (60.1%), with a higher frequency of non-white self-reported ethnicity (47.3%). Most patients (80.1%) were receiving the health services fully financed by the public healthcare system (governmental funding). Patients with a private health insurance coverage were slightly older at diagnosis than those receiving public services (median age of 66.5 years *versus* 63.1 years; *p*=0.013). The smoking status of 74.2% of patients was ‘former smoker’ or ‘current smoker’. Stage IV NSCLC at diagnosis was observed in 82.4% of patients, with 269 of them (68.8%) presenting an adenocarcinomatous histology. Common metastatic sites were the bones (35.1%), pleura (32.3%), and brain (27.0%). The proportion of stage IV patients was significantly higher among adenocarcinoma patients (88.8%) than among squamous cell carcinoma patients (63.2%, *p*<0.05). There was a statistically significance difference (*p*<0.001) between the distribution of histology types in locally advanced and stage IV patients: the proportion of adenocarcinoma (43.5%) was lower than a squamous (46.4%) histology in locally advanced patients, and among stage IV patients, the proportion was 74.2% to 17.1%, respectively.

### EGFR Mutation Testing among metastatic adenocarcinoma patients

Among stage IV patients having adenocarcinoma (n=239), 54.0% were referred for molecular testing, resulting in 129 successfully tested patients (Table 2). For 11 patients (4.6%), this information was not available. The median turnaround time was 13.0 days [range: 2 to 90 days]. The percentage of patients undergoing EGFR mutation testing was significantly higher for patients with private health insurance coverage (70.4% *versus* 52.3%, *p*<0.05).

Among the patients for whom EGFR mutation status was available, 31 (24.0%) were considered to have positive EGFR activating mutations (Table 2). Exon 19 deletion was the most common mutation (48.4%), followed by the L858R mutation in Exon 21 (22.6%). Two patients had double mutations (both T790M and L858R) and three patients (2.3%) had ALK mutations (data not shown). Patients who were never smokers had a significantly higher risk of having positive molecular testing results (51.5% *versus* 9.1% among former or current smokers, *p*<0.001). EGFR activating mutation frequency in stage IV adenocarcinoma was higher (*p*=0.02) in women (33.9%) than in men (16.4%). After binary logistic regression analysis, the difference was not significant between genders, which means that the smoking status may be a confounding factor.

### Treatment patterns

For the 391 enrolled patients, upfront platinum-based chemotherapy was performed in 98 patients (25.1%; 15.9% in stage IIIb and 27.0% in stage IV patients), while non-platinum-based regimens were used in 54 patients (13.8%; 4.3% in stage IIIb and 15.8% in stage IV). Other multimodal treatments were also used, such as a platinum-based chemotherapy associated with radiotherapy of a non-lung site (n=71, 18.2%; 4.3% in stage IIIb and 21.1% in stage IV) or with lung radiotherapy (n=43, 11.0%; 37.7% in stage IIIB and 5.3% in stage IV), and a non-platinum-based regimen associated with non-lung radiotherapy (n=31, 7.9%; 0% in stage IIIB and 9.6% in stage IV) or with lung radiotherapy (n=16, 4.1%; 8.7% in stage IIIb and 3.1% in stage IV). Lung radiotherapy as a single treatment was performed in 18 patients (4.6%; 15.9% in stage IIIb and 2.2% in stage IV) and non-lung radiotherapy as a single treatment was performed in 23 patients (5.9%; 0% in stage IIIb and 7.1% in stage IV). Among all patients, 37 did not receive a treatment based on chemotherapy, radiotherapy, nor targeted therapy (9.5%; 13.0% in stage IIIb and 8.7% in stage IV). The treatment patterns were different between stage IIIb and stage IV patients (*p*<0.001). Among the stage IV adenocarcinoma patients tested for EGFRms (n=129), EGFR-TKIs were prescribed as a first-line treatment in 14 cases; 27 (out of 31) patients with stage IV adenocarcinoma and positive EGFR mutations received an EGFR-TKI treatment (data not shown).

### Survival data

**Figure 1:**
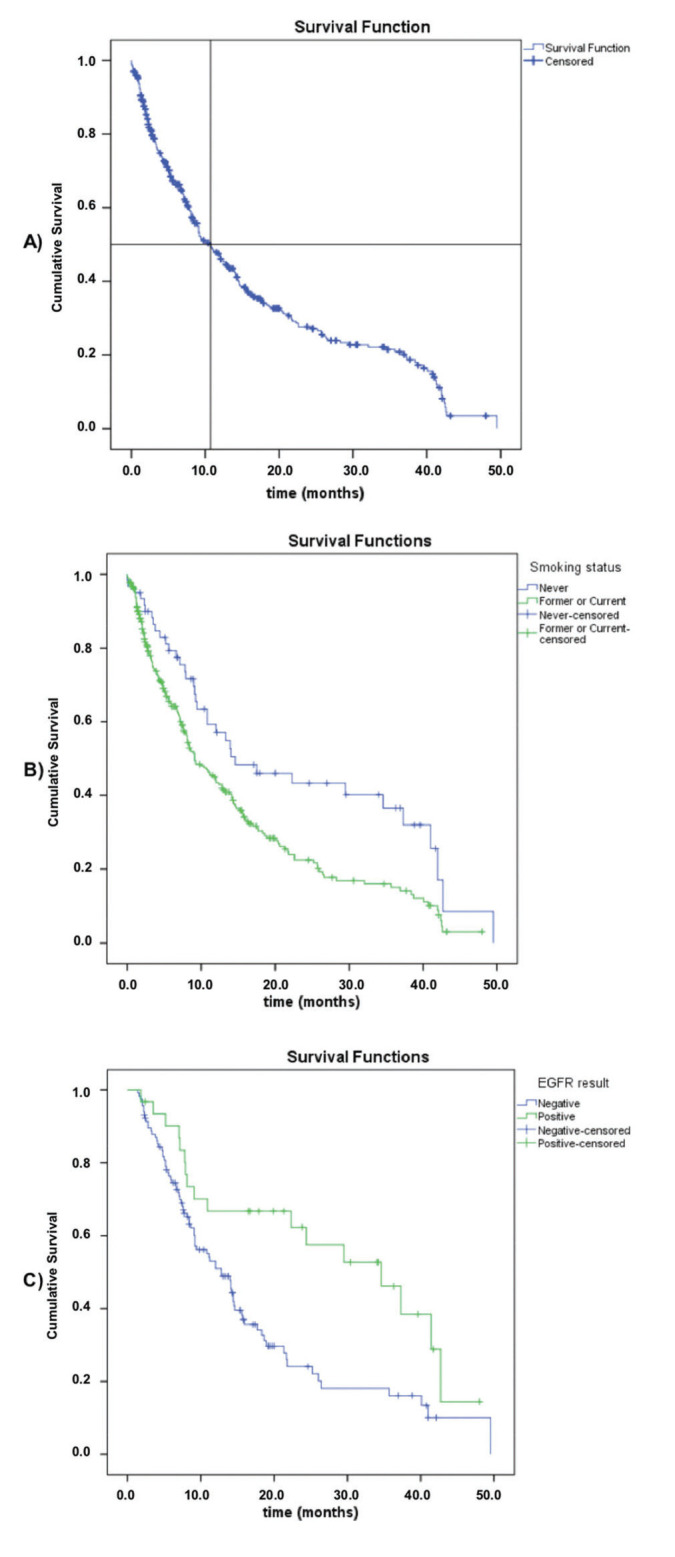
Overall survival data: A) All patients (n=370); B) Based on their smoking status; C) Based on their EGFR mutation status.

Overall survival (OS) data were available for 370 patients, with 248 death events (63.4%) and a median overall survival of 10.8 months (95% CI: 8.5-13.1) (Figure 1). Regarding the smoking status, never smokers had a significantly higher median overall survival compared to current or former smokers (median, 14.6 [95% CI: 4.2-25.0] *versus* 9.1 months [95% CI: 6.9-11.3]; log-rank *p*=0.003). Among the patients subjected to molecular testing (n=147, regardless of the stage or histological type), those with positive EGFRm results had a longer overall survival (median, 34.6 [95% CI: 20.5-48.7] *versus* 12.8 months [95% CI: 9.8-15.8]; log-rank *p*=0.003). There was no difference in OS between the stage IIIb and stage IV groups (log-rank *p*=0.312).

## DISCUSSION

This retrospective analysis of 391 patients from 10 private and public oncology centers depicts an overview of the Brazilian real-world clinical practice in 2014. Our cohort mainly comprised male patients, former or current smokers, with an adenocarcinomatous histology and stage IV disease at diagnosis, receiving public health care services, with a well-balanced ethnicity distribution. These features are consistent with previous analyses of Brazilian patients with NSCLC, except for the histology type, since adenocarcinomatous histology was usually less frequent (7-9). Our data show an opposite trend, with a ratio of adenocarcinoma to squamous cell carcinoma of 3:1 in a locally advanced stage or metastatic NSCLC.

Our findings indicate a significant paucity of molecular testing among metastatic NSCLC patients with adenocarcinoma, with only 54.0% of them being tested for EGFRms. Patients receiving private health care services had higher molecular testing frequencies (over 70%); however, despite the mandatory reimbursement of molecular testing in private health care services at the time of this study, the majority of Brazilian NSCLC patients were still facing significant barriers to access this diagnostic resource. It is worth mentioning that around 75% of the Brazilian population depends solely on public (governmental) funding to access medical care, having restrictions to molecular testing and some specific targeted therapy treatments. Even though our cohort reflects this distribution, the clinical sites from where the participants of this study were enrolled constitute a subgroup of highly complex specialized oncology centers with a better access to health technologies. Therefore, it may not be representative of the overall public oncology practices in the country. However, these results are consistent with the estimates reported in a recent Brazilian literature review (3). Our study revealed that in stage IV adenocarcinoma, the median overall survival was 34.6 months in EGFRm-positive patients who were treated with EGFR-TKIs, 12.8 months in non-EGFRm-positive patients, and 8.0 months (data not shown) in patients who were not subjected to a molecular test. Meanwhile, the lack of molecular testing in stage IV adenocarcinoma may be associated with a shorter overall survival.

An underusage of molecular testing among NSCLC subjects is observed worldwide, such as in the US, Sweden, and Australia (10-13). The 2018 CAP/IASLC/AMP guidelines (4) currently recommend routine EGFR testing for all patients, regardless of their clinical features (such as the stage, smoking status, and histological type, among others). Thus, it is reasonable to expect that testing patterns have changed in recent years in Brazil and that the testing frequency will present higher values in future analyses. The reasons explaining the large proportion of patients not receiving molecular testing in a real-world setting are usually considered multidimensional, involving test costs, turnaround time, the lack of local guidelines reinforcing the need for a routine test, the lack of reimbursement of targeted therapies for positive patients, particularly in the local Brazilian context, in which EGFR-TKIs and EGFR testing were not widely available within the public health care system until 2015 (14).

We observed an EGFR mutation frequency of 24.0% among the adenocarcinoma stage IV patients, in line with previous Brazilian estimates (3), which was higher than that in North America and Europe (for which EGFRm frequencies usually range from 10-20%) and lower than that in Asia (as high as 50%) (15). Its prevalence was also below the global median prevalence (33.1%) (16). The most common mutations reported occurred in Exon 19 (51.6%), followed by Exon 21 L858R (22.6%). Global estimates described by Werutsky et al. indicate a similar trend for Exon 19 mutations, but a significantly higher frequency of mutations in Exon 21 (median, 36.6%; interquartile range, 28.6-47.1%) (16).

In our study, patients who were never smokers had a significantly higher risk of showing positive molecular test results (51.5% *versus* 9.1% among former or current smokers, *p*<0.05). This association was also observed in previous studies (15,16). In 2015, Midha et al. (15) reported differences in the mutation frequency in NSCLC adenocarcinoma patients that were never-smokers, *versus* those that were ever-smokers, according to the geographic region and found the following values: Europe, 35% *versus* 8%; Asia-Pacific, 64% *versus* 33%; Indian subcontinent, 32% *versus* 17%; Africa, 41% *versus* 6%; and North America, 47% *versus* 14%. Despite the solid evidence pointing to a higher chance of a EGFRm positivity among never-smokers in the literature, the smoking status has not been considered a criterion used to decide whether patients should or should not receive molecular testing anymore (4,17). Initial observations showing that the majority of lung cancer patients without a previous history of smoking presented EGFRm positivity led some clinicians and policy decision-makers, worldwide, to adopt this testing exclusion criterion (17). However, the growing knowledge regarding driver mutations in NSCLC and their interaction with other exposure variables subsequently modified this understanding.

With regard to the treatment patterns, the majority of patients (54.2%) in our cohort received a first-line platinum-based systemic therapy, whether alone or in combination with radiation therapy. These findings are consistent with the observations made by Younes et al. in 2011 (18) and Naime et al. in 2007 (19) for NSCLC cases treated in Brazil in the 1990 and 2000 decades, perhaps indicating that the treatment patterns for lung cancer have been steady in the country for the last 3 decades. Most patients were treated in a public health care system context, in which the incorporation and reimbursement of novel therapies for cancer patients face significant barriers, resulting in a delayed access of treatments for patients. EGFR-TKIs were only made available within the public health care system in 2015, but molecular testing and medications are still provided inconsistently across the country, impairing eligible patient identification and the availability of drugs. This probably explains the lower proportion of patients receiving EGFR-TKIs as a first-line treatment in our cohort.

A review conducted by Araújo et al. described that the median OS ranges from 5.3 to 62.9 months in Brazilian cohorts of NSCLC metastatic patients, depending on the systemic therapy that patients had received and on the setting in which they were treated (private or public). Studies with larger cohorts (n=205 to 2,673) had a median OS ranging from 5.3 to 12.2 months, similar to our findings (10.8 months). The factors associated with an improved OS were: being a never smoker and showing positive EGFR molecular testing results, similar to those of other analysis (17,20).

The strengths of this study include the presence of a real-world setting, the inclusion of patients with both private and public coverage, and the simultaneous analysis of molecular testing and treatment patterns and also the survival in the same cohort, providing relevant information about the Brazilian NSCLC patients for both care providers and policy decision-makers. This is especially relevant for the public health care system, in which barriers to access molecular testing and targeted therapies seem to be markedly present, leading to significant unmet needs for patients.

This study also has some limitations. We were not able to investigate a broader set of variables potentially linked to EGFRm testing patterns that could explain the reduced proportion of the tested patients. The small cohort size of tested patients and the low likelihood of using sensitive methods for uncommon EGFR mutations in this cohort may have also impaired our ability to derive robust estimates of the prevalence of specific mutations, to identify more atypical, but clinically relevant mutations, and to observe mutation rates among subgroups of patients (according to their ethnicity, for example). In the present study, diagnosis and treatment were conducted between 2014 and 2017; thus, changes in molecular testing and treatment patterns are expected to have happened during the recent years. Finally, the availability of EGFR-TKIs, even in the public health care system, is aligned with the reinforcement that EGFRm testing relevance may impact NSCLC treatment and its respective outcomes. This perspective supports the need for further generating local data in the upcoming years.

## CONCLUSIONS

In conclusion, the growing body of knowledge regarding activating mutations in lung cancer and the enrichment of the therapeutic armamentarium for EGFRm-positive patients have the potential to transform the natural history of the disease. Although we expect different testing and treatment patterns in the current Brazilian context, our findings provide relevant information for prescribers and policy decision-makers by highlighting the unmet needs of patients, the importance of molecular testing in newly diagnosed locally advanced or metastatic lung adenocarcinoma with the respective EGFR-TKI treatment when EGFRms are present, and the areas in which further efforts are required to grant access to effective treatment. 

## AUTHOR CONTRIBUTIONS

All authors affirm that they have contributed to the development of the manuscript, were involved in all stages of the development, and have approved the submitted manuscript.

Marinho F and De Marchi P collected the data, evaluated the study findings, discussed the results and conclusions and wrote the manuscript. Cronemberger E, Baldotto C, Araújo LH, Almeida T, Calabrich A, Salles P and Franke F collected the data, evaluated the study findings, discussed the results and conclusions and wrote the manuscript.

Custodio MG contributed to the study conceptualization, formal analysis, methodology, and project administration. He also supervised and provided support in the writing of the original draft, review, and editing. Maria AS and Pereira MH contributed to the formal analysis and project administration. He also supervised and provided support in the writing of the original draft, review, and editing. Jr GC conceived and designed the study, contributed to data collection and data analysis, verified the analytical methods, evaluated the study findings, discussed the results and conclusions and wrote the manuscript.

## Figures and Tables

**Table 1:** Baseline Demographic and Clinical Characteristics (n=391).

Characteristic	N (%)
Sex - male	235 (60.1)
Age, years (median [min-max])	64.1 [23.7-98.7]
Self-reported ethnicity	
Non-White	185 (47.3)
White	164 (41.9)
Asian	1 (0.3)
Unknown	41 (10.5)
Health services funding	
Public (government)	313 (80.1)
Health insurance/private	78 (19.9)
Smoking status	
Former or current	290 (74.2)
Never	63 (16.1)
Unknown	38 (9.7)
Histological type	
Adenocarcinoma	269 (68.8)
Squamous cell carcinoma	87 (22.3)
Large cell carcinoma	11 (2.8)
Mixed	2 (0.5)
Unknown	22 (5.6)
Stage	
IIIb	69 (17.6)
IV	322 (82.4)
Metastasis site† (n=322)	
Bones	113 (35.1)
Pleura	104 (32.3)
Brain	87 (27.0)
Lymph nodes	73 (22.7)
Adrenal glands	47 (14.6)
Liver	40 (12.4)
ECOG Performance Status	
0	15 (3.8)
1	164 (41.9)
2	69 (17.6)
3	47 (12.0)
4	9 (2.3)
Unknown	87 (22.3)

† Pulmonary metastasis was not captured in this study; ECOG, Eastern Cooperative Oncology Group.

**Table 2:** Distribution of EGFR mutation types among adenocarcinoma stage IV patients.

Distribution of EGFR mutations	Number of patients (%)
Patients tested	129 (54.0)
Negative EGFR activating mutation	98 (76.0)
Positive EGFR activating mutation	31 (24.0)
Exons	
Exon 18	2 (6.5)
G719X	1 (3.2)
G721D	1 (3.2)
Exon 19	16 (51.6)
Deletion	15 (48.4)
Other mutation in Exon 19	1 (3.2)
Exon 20	6 (19.3)
Insertion	2 (6.5)
T790M and L858R (double)	2 (6.5)
Other mutation in Exon 20	2 (6.5)
Exon 21	7 (22.6)
L858R	7 (22.6)
